# The Germline Targeting Vaccine Concept: Overview and Updates from HIV Pre-Clinical and Clinical Trials

**DOI:** 10.2174/011570162X358302250206074255

**Published:** 2025-02-21

**Authors:** Leonidas Stamatatos

**Affiliations:** 1 Vaccine and Infectious Disease Division, Fred Hutchinson Cancer Research Center, Seattle, WA, 98109, USA

**Keywords:** Broadly neutralizing antibodies, HIV, VRC01-class, 426c, germline targeting, vaccines

## Abstract

An effective HIV-1 vaccine should elicit diverse immune responses, including broadly neutralizing antibodies (bNAbs). Such antibodies recognize regions of the viral envelope glycoprotein (Env) that are conserved among the diverse HIV-1 clades and strains. They are isolated from people living with HIV-1 to protect animals from experimental viral exposure and reduce HIV-1 acquisition in clinical settings. However, despite efforts spanning several decades, bNAbs have not been elicited through immunization. The HIV Env efficiently binds bNAbs, but not their unmutated (germline, gl) precursors. In contrast, Env readily engages the germline precursors of antibodies with no, or very narrow, cross-neutralizing activities (non-neutralizing antibodies, nnAbs). That, in part, explains why Env-based immunogens consistently elicit nnAbs, but not bNAbs. In the past decade, Env-derived proteins have been specifically designed to engage the germline precursors of diverse bNAbs. These ‘germline-targeting’ Env immunogens activate the corresponding naive B cells in vivo, but are unable to guide their proper maturation towards their broadly neutralizing forms. For this, immunizations with currently not well-defined heterologous Envs are required. Here, we discuss the development of germline-targeting Env immunogens, their *in vivo* evaluation, and the strategies currently under evaluation that aim to rapidly guide the maturation of germline-precursor BCRs into their broadly neutralizing forms.

## 1 INTRODUCTION

Broadly neutralizing HIV-1 antibodies (bNAbs) prevent infection by diverse HIV-1 strains because they recognize conserved epitopes on the viral envelope glycoprotein (Env). Broadly neutralizing antibodies (bNAbs) could be an important component of the diverse anti-viral immune responses an effective vaccine should elicit [1-5]. However, despite extensive efforts, over the past three decades, bNAbs have not been elicited through immunization of wild-type animal species or humans (except in very few rare cases). These efforts include the testing of Envs derived from different viral strains/clades as immunogens, different forms of Env (such as monomeric, trimeric, soluble, and membrane-bound), different adjuvants, and different immunization platforms (including recombinant protein, DNA, and mRNA).

Here, we discuss the main obstacles facing the development of bNAbs that target the receptor binding site of Env (CD4-binding site, CD4-BS) through vaccination, how knowledge of the ontogeny and structures of newly isolated bNAbs has led to the development of new hypotheses on how to elicit such antibodies through immunization with optimized immunogens, and how these immunogens and vaccine concepts are currently tested and validated in preclinical and clinical settings. Anti-Env antibodies, including bNAbs, display Fc-mediated effector functions, which may also contribute to controlling infection [6-10], but here, we focus on the neutralizing functions of anti-HIV-1 antibodies.

### 1.1 Development of Broadly Neutralizing HIV-1 Antibody Responses During HIV-1 Infection

Cross-neutralizing HIV-1 antibody activities are detectable in sera from people living with HIV-1 (PLWH). Cross-neutralizing means the ability of serum antibodies to neutralize not only the autologous virus, but heterologous viruses as well. The breadths (how many distinct viruses are neutralized by a serum sample) and potencies of serum cross-neutralizing activities vary widely [11-19], and only a small fraction of PLWH develop exquisitely potent and broad anti-HIV-1 neutralizing antibodies [17]. Several factors (such as the duration of infection) have been associated with the development of cross-neutralizing serum antibody responses [11, 13, 14, 20, 21]. Potent cross-neutralizing antibody responses remain detectable even during prolonged periods of antiretroviral therapy (ART) [22].

Until 2010, only a handful of bNAbs were discovered [23-27], but the development of high-throughput HIV-1 neutralization assays, optimal Env-derived reagents, and high-throughput VH/VL gene-sequencing techniques led to the isolation of a large number of new bNAbs with impressive neutralization breadths and potencies. Detailed characterization of the ontogeny of these bNAbs, their epitopes, and their structures has vastly improved our understanding of how bNAbs are generated during infection and how they engage their epitopes on the trimeric Env to prevent viral entry into target cells.

### 1.2 Broadly Neutralizing Antibodies can Reduce HIV-1 Acquisition

The neutralizing activities of anti-HIV1 antibodies are determined using well-established, high-throughput neutralization assays [19, 28, 29]. The *in vivo* preventive potential of HIV-1 bNAbs has been demonstrated in appropriate animal models by passive administration, or *in vivo* expression, of a broadly neutralizing monoclonal antibody (mAb), prior to viral exposure. In the case of non-human primates, a chimeric virus that expresses an HIV-1 Env and the SIVmac background [S(H)IV] [30-45] has been used, while in the case of humanized mice, HIV-1 has been employed [36, 46, 47]. Such studies have demonstrated that bNAbs of diverse epitope specificities can protect animals from infection and that the mAb concentration correlates with the level of protection.

So far, only one broadly neutralizing mAb (VRC01) has been evaluated in phase 3 clinical trials (HVTN 703/704) and shown to limit acquisition by sensitive viruses [48]. The fact that mAb VRC01 was effective against sensitive viruses (i.e., viruses that readily express its epitope), it was expected that it would not escape the viruses that have acquired mutations that directly or indirectly alter the conformation or exposure of that epitope on the viral Env. The results from this critical study have justified vaccines’ efforts to elicit bNAbs, but they have also clearly indicated that bNAbs of diverse epitope specificities must be co-elicited by an effective HIV vaccine to prevent infection from diverse circulating HIV-1 strains. mAb VRC01 belongs to a class of anti-CD4-BS antibodies termed VRC01-class [49-55]. Additional anti-CD4-BS broadly neutralizing mAbs (with greater potencies than VRC01), along with bNAbs that bind to other conserved epitopes on Env, are currently under clinical investigation.

### 1.3 Why are not Broadly Neutralizing Antibodies Generated by Vaccination?

The epitopes of known bNAbs are located within the receptor binding site CD4-BS [56-58], the apex of the Env trimer [59-64], an area around the conserved N332 glycan site in V3 [65-71], a cluster of mannose residues on gp120 [26, 72-74], the fusion peptide of the gp41 subunit [75], the extracellular part of the transmembrane gp41 subunit [25, 76, 77], and the gp120-gp41 interface [27, 78, 79].

Diverse Env proteins are readily recognized by the known bNAbs, which means that their epitopes are present and available on Env. One can, therefore, expect that when used as immunogens, such Envs could readily activate B cells that express B cell receptors (BCRs) that bind those epitopes, and that those B cells could eventually produce such bNAbs. The question thus arises as to why Env protein immunogens do not (or very rarely) elicit bNAbs.

### 1.4 Obstacles Facing the Elicitation of VRC01-class Antibodies Through Vaccination

Here, we discuss the efforts to elicit a particular class of anti-CD4-BS bNAbs, called ‘VRC01-class’, as an example to illustrate some of the obstacles facing the development of bNAbs in general by immunization.

VRC01-class of bNAbs are all derived from the pairing of LCs expressing rare 5 amino acid-long CDRL3 regions with HCs derived from the VH1-2*02 or *04 alleles. These antibodies have been isolated from several PLWH and are highly mutated from their gene-encoded sequences [49-55, 80, 81]. In fact, they can be up to 50% divergent from each other, but despite this significant amino acid sequence divergence, they adopt similar structures and recognize the same epitope within the CD4-BS, with very similar angles of approach. In contrast to most antibodies, VRC01-class antibodies primarily engage their target epitope through the gene-encoded CDRH2 domains [54, 55, 82-86]. It is important to note, however, that not every human BCR formed by the above-mentioned VH/VL combinations can target the CD4-BS. For example, the human antibody FH1, which was isolated from a participant of the HVTN100 clinical trial, expressed an HC derived from the VH1-2*02 allele and a light chain commonly found in VRC01-class bNAbs (κ3-20) expressing 5 amino acid CDRL3, but instead of binding to the CD4-BS, it recognized the C1C2 domain of gp120 and did not neutralize HIV-1 [87]. Mutations in the CDRH3 and CDRL3 of FH1 altered its epitope specificity so that it binds to the CD4-BS. Thus, although most of the contacts made by VRC01-class antibodies with the CD4-BS were through their gene-encoded CDRH2 regions, their CDRH3 regions also contributed to their binding to Env [85].

**Fig. (1):**
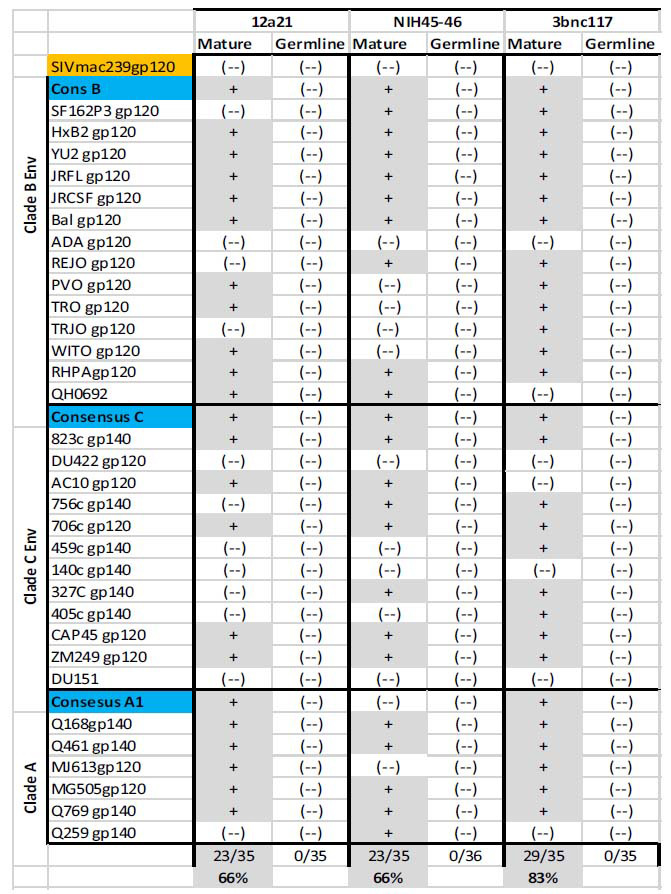
Diverse Envs are recognized by the mature forms of VRC01-class bNAbs, but not by their unmutated forms. Recombinant Env proteins (gp120 or gp140 forms) from different clades were evaluated for recognition (by ELISA) by the mature and germline (unmutated) forms of three VRC01-class antibodies: 12a21, NIH45-46, and 3bnc117. SIVmac239gp120 was used as an internal negative control. (+): binding. (-): no binding.

It is now well established that while diverse Envs are readily recognized by the mutated (broadly neutralizing) forms of VRC01-class antibodies, they are not recognized by the corresponding germline antibody forms [88-91] (Fig. **1**). This is not unique to VRC01-class antibodies. The first report that Envs do not engage the germline forms of bNAbs was published by X. Xiao *et al.* in 2009, at a time when only a few cross-neutralizing antibodies were known (such as b12, 2G12, and 2F5) [92]. Those early observations led D.S. Dimitrov to propose that naive B cells expressing the unmutated precursors of bNAbs could be activated by using immunogens that may be different than the Env, but capable of binding to the germline antibodies, *i.e*., to serve as primary immunogens [93].

**Fig. (2):**
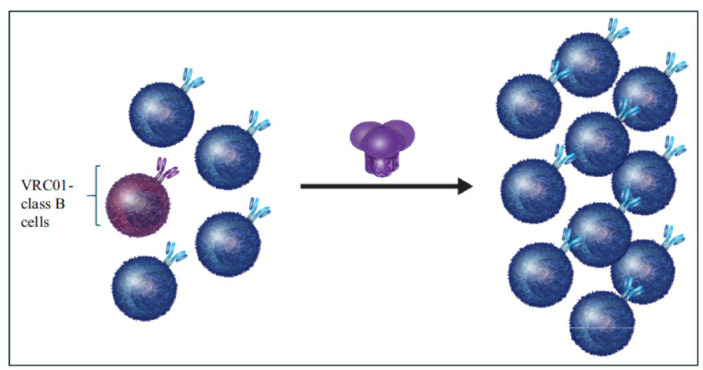
Env immunogens fail to activate naive B cells expressing VRC01-class BCR precursors. The unmutated forms of VRC01-class bNAbs do not bind their epitope on HIV-1 Envs. When such Envs are used as immunogens, they do not activate naive B cells expressing the unmutated BCR forms of VRC01-class antibodies, but they efficiently activate naive B cells expressing the unmutated BCR forms of non-neutralizing antibodies.

Thus, one major reason why Env immunogens do not elicit VRC01-class bNAbs is due to their inability to engage and activate the naive B cells that have the potential of producing VRC01-class bNAbs, *i.e.*, the first step in the production of such antibodies. In contrast to the lack of interaction between the germline forms of VRC01-class bNAbs and Env, non-neutralizing antibodies (nnAbs) readily engage diverse Envs [90]. As a result, during Env immunization, naive B cells expressing the germline BCR forms of nnAbs become readily activated while those expressing the germline forms of VRC01-class bNAbs do not (Fig. **2**).

The observation that germline forms a group of antibodies with very similar ontogeny that do not bind Env and in order for the corresponding naive B cells to be activated, specifically designed proteins (germline-targeting) have to be designed, which can form the basis of the ‘germline-targeting’ immunization concept. Importantly, as natural Envs that guide the maturation of germline VRC01-class antibodies towards their broadly neutralizing forms during HIV-1 infection are also unknown, such immunogens must be developed and validated experimentally.

### 1.5 The Development of Germline-targeting Immunogens to Engage Germline VRC01-class B Cell Receptors

#### 1.5.1 Clade C-derived gl-targeting Env (426c)

In 2013, while investigating the interaction of VRC01-class bNAbs with Env [88, 94], we reported that the elimination of three N-linked glycosylation sites (NLGS), one in loop D (N276) and two in V5 (N460 and N463) from the clade C Env 426c (426c triple mutant, 426c.TM), allowed some, but not all known glVRC01-class antibodies, to bind to it [94]. The unmutated form of the 426c Env was only recognized by the mature VRC01-class antibodies, like all the other Envs we examined.

The variable domains V1, V2, and V3 on the gp120 subunit were positioned in such a way as to limit the accessibility of the CD4-BS and their removal increased the exposure of the CD4-BS to antibody-binding and neutralization by anti-CD4-BS antibodies [95, 96]. The partial deletion of V1, V2, and V3 from 426c.TM (426c.TM4ΔV1-V3; now referred to as 426c.Mod.Core) resulted in its recognition by an increased number of known glVRC01-class antibodies (as compared to 426c.TM) and with greater affinities [91]. 426c.Mod.Core has been extensively evaluated preclinically and it is being evaluated in a phase 1 clinical trial (HVTN301; ClinicalTrials.gov; ID: NCT05471076).

#### 1.5.2 Clade B-derived germline-targeting Env

Employing a different approach to ours, J. Jardine and colleagues redesigned the outer domain (eOD) of the clade B Env HxB2 gp120 to bind with high affinity to most of the known glVRC01-class antibodies [89]. Additional improvements were subsequently made to further increase the overall affinity of this protein for glVRC01-class antibodies. One such optimized version (eOD-GT8) has been extensively evaluated in animal models and in a phase 1 clinical trial (G001, ClinicalTrials.gov; ID: NCT03547245) [97].

#### 1.5.3 Clade A-derived targeting Env

In 2017, Medina-Ramirez *et al.* reported the design of soluble trimeric Env protein derived from the clade A BG505 virus (GT1) that bound glVRC01-class antibodies [98]. It lacked the key NLGSs in loop D (N276) and V5, as discussed above, but also had additional mutations (19 in total) that were introduced to improve the stability of the trimeric Env and its recognition by glVRC01-class antibodies. GT1 activated B cell lines engineered to express the germline forms of some of the known VRC01-class antibodies and activated naive B cells expressing germline forms of VRC01-class BCRs in a KI mouse model. Additional modifications were introduced on the GT1 background to further optimize the ability of this Env to engage glVRC01-class antibodies (termed, GT1.1 and GT1.2). GT1.1 is currently being evaluated in a phase 1 clinical trial (C101; ClinicalTrials.gov; ID: NCT04224701).

### 1.6 Germline-targeting Envs Activate B Cells Expressing Germline VRC01-class B Cell Receptors *in vitro*

To optimize the B cell activation abilities of 426c.Mod.Core and eOD-GT8, these proteins were oligomerized; eOD-GT8 was expressed as a 60mer on the surface of lumazine synthetase [99], while 426c.Mod.Core was expressed as self-assembling nanoparticles (5-7 molecules per nanoparticle) by the addition of the C-terminal oligomerization domain of the C4b’s α-chain [91, 100]. Due to its trimeric configuration, GT1.1 is capable of activating B cells without additional oligomerization [98].

The abilities of these germline-targeting proteins to activate B cells expressing germline forms of VRC01-class antibodies have been demonstrated first *in vitro* using B cell lines engineered to express the corresponding BCRs [89, 94, 98, 101, 102]. Such experiments have also demonstrated that upon activation, the germline-targeted immunogens become internalized [90], and it is, therefore, expected that the activated B cells will present Env peptides on their surface and receive the necessary T cells required for their survival.

### 1.7 Testing Germline-targeting Env Immunogens *in vivo*

Several wild-type (WT) animal species (such as mice, rats, rabbits, or NHPs) are not suitable for testing immunogens aiming to produce VRC01-class bNAbs, as they do not express orthologs of the human VH1-2*02 or *04 alleles [103]. Transgenic mice have been engineered to express different forms of glVRC01-class BCRs [91, 101, 102, 104-112].

These mouse models have been found to be instrumental in validating the critical importance of germline-targeting immunogens for the activation of naive B cells expressing glVRC01-class BCRs. Indeed, 426c.Mod.Core, eOD-GT8, and GT1.1 can readily activate naive B cells expressing such BCRs after a single immunization and the activated B cells enter the GC reaction where they accumulate somatic mutations over extended periods of time [91, 101, 102, 104-110]. These mutations improve the Env recognition properties of the elicited VRC01-class antibodies.

The frequencies of B cells expressing VRC01-class BCRs in these KI mice vary (between 0.08% and ~40%), and are, therefore, higher than those present in humans (0.002%-0.0002%) [99, 113, 114]. To examine whether germline-targeting immunogens can activate glVRC01-class B cells when present at near physiological frequencies, VRC01-class B cells from the transgenic mice were transferred into WT mice at decreasing numbers. The WT animals were then immunized with germline-targeting immunogens. The frequencies of the transferred glVRC01-B cells in the lymph nodes and spleens of the WT animals were determined at different times after immunization [112, 115-117]. These studies revealed that both the frequencies of the transferred target B cells and the affinities of the germline-targeting immunogens regulated the efficiency by which germline-targeting immunogens activated and recruited the target B cell clones in the germinal centers. These studies were conducted with the adjuvanted recombinant protein forms of germline-targeting immunogens, but recent data indicate that germline-targeting immunogens can very effectively activate the targeted B cells when delivered through mRNA-based platforms as well [118].

### 1.8 Germline-targeting Immunogens Elicit Partially Mutated VRC01-class Antibodies with Different Binding Potentials

One could expect that partially mutated VRC01 antibodies elicited by one germline-targeting immunogen would be able to bind to the other germline-targeting immunogens. This does not, however, appear to always be the case. Immunization with 426c.Mod.Core elicited partially-mutated VRC01 antibodies that could bind not only to 426c.Mod.Core, but also eOD-GT8 [108], and as expected, VRC01-class B cells activated by 426c.Mod.Core could be identified and isolated using eOD-GT8-based tetramer probes [108, 109]. Immunization with eOD-GT8, however, elicited partially mutated VRC01 antibodies that efficiently bound to eOD-GT8, but not to 426c.Mod.Core [108]. As discussed above, the former expressed both the outer and inner gp120 domains, while the latter was based only on the outer gp120 domain. The presence of the inner domain on 426c.Mod.Core may have restricted the binding of the VRC01-class antibodies elicited by eOD-GT8. These observations imply that VRC01-class antibodies elicited by the different germline-targeting Envs can recognize the VRC01 epitope with slightly different orientations. The additional steric restrictions imposed by glycans surrounding the CD4-BS and the positioning of the V1, V2, and V3 domains on trimeric Envs may allow only a subfraction of the antibodies elicited by 426c.Mod.Core and eOD-GT8 to engage trimeric Envs.

### 1.9 Clinical Evaluation of Germline-targeting Immunogens

eOD-GT8 has been evaluated in humans in a phase 1 clinical trial as a nanoparticle (lumazine synthase) adjuvanted with the AS01_B_ adjuvant (G001 clinical trial) [97]. Detailed analysis of the elicited antibody and B cell responses clearly indicated VRC01-class B cells to be activated in almost all participants and that the activated B cells accumulated somatic hypermutations (SHMs) in their BCRs over time. Importantly, the results have been found to be in support of the above-discussed ‘germline-targeting’ vaccine concept, as they demonstrated that Env-derived proteins can be specifically designed to engage specific germline bNAb BCRs and that they do exactly that when employed as immunogens in clinical settings. As mentioned above, the other two germline-targeting immunogens, 426c.Mod.Core-C4b (HVTN301; ClinicalTrials.gov; ID: NCT05471076) and GT1.1 (C101; ClinicalTrials.gov; ID: NCT04224701) are currently under clinical evaluation, and the preliminary results seem promising.

The above clinical trials have been conducted involving participants not infected with HIV-1. That is, there has been no pre-existing antibody response to the germline-targeting immunogen. To examine whether the ability of the 426c.Mod.Core-C4b to activate naive B cells expressing germline VCR01-class BCRs was affected (positively or negatively) or not by pre-existing anti-Env antibody responses, we have recently initiated a phase 1 clinical trial involving PLWH on ART (HVTN807, ClinicalTrials.gov; ID: NCT06006546). In HVTN807, we will also examine the interplay between pre-existing (i.e., virus-elicited) and vaccine-induced B cell and antibody responses and the rebounding virus during anti-retroviral treatment interruption [119].

### 1.10 Heterologous Env Booster Immunizations are Required for the Proper Maturation of VRC01-class Antibody Responses

For the germline forms of VRC01-class antibodies to become broadly neutralizing, they have to accumulate somatic mutations in both their VH and VL domains. As mentioned above, the known VRC01-class bNAbs are extensively mutated and their amino acid sequences can be up to 50% divergent from each other. Not all the mutations that VRC01-class bNAbs accumulate in the context of infection are required for their broad neutralizing activities, but some of them are critical because they allow the antibody to bypass the key obstacles it is facing on Env, such as the glycans expressed on the conserved N276 NLGS in loop D [120, 121].

**Fig. (3):**
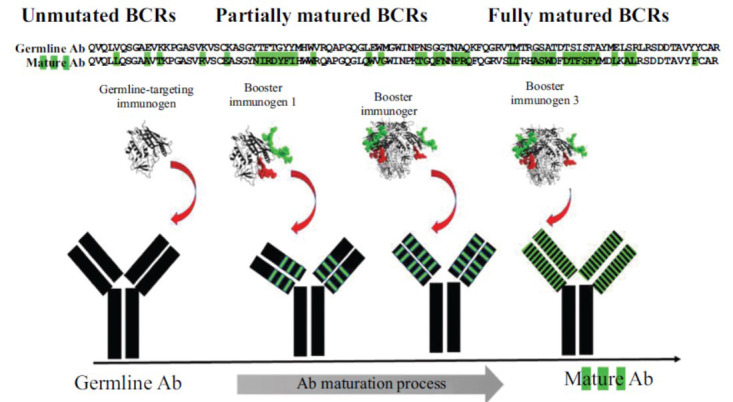
Guided maturation of a VRC01-clas bNAb precursor during prime-boost immunizations. To elicit VRC01-class bNAbs through immunization, the first immunogen activating the glBCR of interest (‘germline-targeting’ immunogen) and heterologous immunogens (‘booster’ immunogens) would be administered in a specific order to select and further activate those BCRs that have accumulated appropriate mutations (green). The amino acid sequences, germline and mature, of a VRC01-class antibody are shown. Green and red colored glycans represent those expressed at position N276 in loop D (red) and in V5 (green).

Once activated by a germline-targeting immunogen, naive VRC01-class B cells can enter the germinal center reaction [122] where their VH/VL genes accumulate random nucleotide mutations. In some cases, these mutations can lead to amino acid changes. As a result, daughter memory B cells expressing VRC01 BCRs with different amino acid sequences co-exist in the same animal/human. Some, but not all, daughter B cells may express VRC01 BCRs with mutations enabling them to accommodate N276-glycans. However, because the current germline-targeting immunogens lack that specific NLGS, repeat immunizations with a germline-targeting immunogen do not select for those BCRs capable of bypassing the N276-associated glycans [101]. Hence, it has been proposed that immunizations with specifically selected heterologous Envs (‘booster’ immunogens) are required for the maturation of germline VRC01 BCRs toward their broadly neutralizing forms [101, 102, 104, 106, 109] (Fig. **3**).

The key requirement of the ‘booster’ Env is to select those daughter cells whose BCRs have accumulated mutations that allow them to overcome an obstacle that has been absent from the germline-targeting immunogen. For example, in the case of the 426c.Mod.Core, expressing both the inner and outer gp120 domains, but lacking the N276- and V5-associated NLGS and variable loops V1/V2/V3, the first boost immunogen could ideally be one that would select BCRs with mutations that allow them to bypass the N276- and V5-associated glycans on heterologous gp120 cores. Indeed, immunization with 426c.Mod.Core gave rise to VRC01 BCRs that could bypass the N276- and V5-associated glycans on some, but not all, heterologous gp120 cores [109]. Immunization with one such heterologous immunogen (HxB2.WT.Core) stimulated the daughter B cells expressing such BCRs [109, 110]. However, a third immunization with a cocktail of trimeric Envs resulted in the development of VRC01 antibodies that could efficiently neutralize diverse viruses whose Envs lacked the N276 NLGS, but still not viruses expressing N276 glycans [123].

In the case of eOD-GT8, which only expresses the outer gp120 domain, the first booster immunogen could be one that selects those BCRs that can bind the VRC01 epitope in the presence of the inner gp120 domain, and ideally the N276- and V5-associated glycans as well. Indeed, eOD-GT8 elicited BCRs that could recognize heterologous core gp120s, *i.e*., they could bypass the inner domain when the N276 glycans were absent [107]. Booster immunization with one such heterologous core (BG505 core-GT3) expanded these BCRs, and a third immunization with a stabilized Env trimer that also lacked the N276 glycans (BG505 N276D) further expanded the VRC01 B cells that could bypass most of the obstacles presented by the full Env, but not the N276-associated glycans [104]. As a result, these VRC01 antibodies can neutralize diverse viruses whose Envs lack the N276 NLGS. More recently, Cottrel, C. A. *et al.* reported that a prime immunization with eOD-GT8, followed by a heterologous boost immunization with a core gp120 protein lacking the N276 glycan (core-g28v2), is sufficient to elicit VRC01 antibodies that can neutralize viruses whose Env lack the N276 glycans [123].

Similarly, GT1.1 and GT1.2 could activate native B cells expressing the unmutated forms of VRC01-class antibodies in KI mice. Booster immunizations with heterologous trimeric Envs lacking the N276 NLGS, followed by immunizations with fully glycosylated trimeric Envs, resulted in the isolation of VRC01 antibodies displaying neutralizing activities against heterologous viruses whose Envs lacked the N276 glycans, but not fully glycosylated Envs [105].

In one study, however, Chen, C. H. *et al.* reported that cross-neutralizing VRC01-class antibodies were elicited through a prime-boost immunization schema employing 9 different Envs and lasting ~2 years [106]. Encouragingly, characterization of the elicited VRC01-class antibodies revealed that they had accumulated some of the key mutations present in human VRC01-class bNAbs.

## CONCLUSION

The recently gained knowledge on how broadly neutralizing antibody responses develop during chronic HIV-1 infection, in particular, how specific B cell clones become activated and how their BCRs evolve to adopt broadly neutralizing forms, have led to the development of Env-derived molecules that can efficiently activate naive B cells expressing the precursor BCR forms of bNAbs. Based on these findings, two major immunization approaches have been developed, the ‘germline-targeting’ and the ‘lineage’. Both approaches are currently being evaluated in multiple phase 1 clinical trials and the initial results are very encouraging as they demonstrate that germline-targeting Env immunogens can initiate the B cell maturation processes, leading to the emergence of bNAbs. This process, however, requires several follow-up immunizations with heterologous Envs. Also, booster immunogens that could guide the maturation of VRC01 antibodies toward their complete broadly neutralizing forms remain to be determined.

## AUTHORS’ CONTRIBUTIONS

The author confirms sole responsibility for the following: study conception and design, data collection, analysis and interpretation of results, and manuscript preparation.

## LIST OF ABBREVIATIONS

Ab: Antibody
ART: Antiretroviral therapy
ATI: Analytical treatment interruption
bNAb: Broadly neutralizing antibody
CDR: Complementarity-determining region
CD4-BS: CD4-binding site
Env: Envelope glycoprotein
HVTN: HIV vaccine trials network
KI: Lnock-in
nnAb: Non-neutralizing antibody
mAb: Monoclonal antibody
BCR: B cell receptor
HIV-1: Human immunodeficiency virus type 1
HC: Antibody heavy chain
SIV: Simian immunodeficiency virus
LC: Antibody light chain
gl: Germline
N: Asparagine
D: Aspartic acid
VH: Variable heavy chain domain
VL: Variable light chain domain
